# Evaluating Challenges in Access To Transplantation for Persons with HIV

**DOI:** 10.1007/s11904-025-00735-2

**Published:** 2025-03-21

**Authors:** Ruth O. Adekunle, Moreno Rodrigues, Christine M. Durand

**Affiliations:** 1 Division of Infectious Diseases, Medical University of South Carolina, 135 Rutledge Avenue, 12th Floor, MSC 752, Charleston, SC 29425 USA; 2 Department of Medicine, Johns Hopkins University School of Medicine, 2000 E. Monument Street Room 103, Baltimore, MD 21205 USA

## Abstract

**Purpose of Review:**

Antiretroviral therapy has significantly improved the life expectancy of people with HIV (PWH), leading to an increased prevalence of comorbidities such as end-stage organ diseases. PWH with end-stage disease face a significantly higher risk of mortality compared to those without HIV, highlighting the urgent need to improve access to organ transplantation for this vulnerable group. This review examines barriers to organ transplantation for PWH, utilizing a modified five A’s model (acceptability, availability, accessibility, affordability, accommodation).

**Recent Findings:**

Despite comparable post-transplant outcomes to the general population, PWH are less likely to receive organ transplants. The HIV Organ Policy and Equity (HOPE) Act has expanded the donor pool by permitting organ transplants from donors with HIV to recipients with HIV. However, factors limiting expansion include policy, logistical constraints, and HIV-related stigma.

**Summary:**

Despite pivotal advancements in HIV organ transplantation, multilevel challenges continue to limit access for PWH. Addressing these barriers is essential to ensuring equitable access to this life-saving therapy.

## Introduction

Advancements in antiretroviral therapy (ART) have allowed for improved survival among people with HIV (PWH). With advancing age, PWH are at higher risk for conditions such as hypertension, diabetes, cardiovascular disease, metabolic-dysfunction-associated steatotic liver disease, and pulmonary arterial hypertension, which contribute to an elevated risk of end-stage organ diseases [1]. In fact, PWH are now more likely to die from end-organ diseases than from opportunistic infections [2].

Historically, HIV was considered a contraindication for organ transplantation, as most PWH progressed to AIDS and often succumbed to organ failure within months of diagnosis [3]. The ethical acceptability of organ transplantation in PWH was questioned due to the scarcity of donor organs. Additionally, there were significant concerns that the necessary immunosuppression would increase the risk of opportunistic infections, potentially accelerating mortality [3]. However, data from landmark studies have since demonstrated that organ transplantation is not only feasible for PWH but also the preferred treatment for organ failure [4–6]. Despite these advancements, PWH still face disparities in access to transplantation and are less likely to be waitlisted and receive an organ transplant [7, 8].

**Fig. 1:**
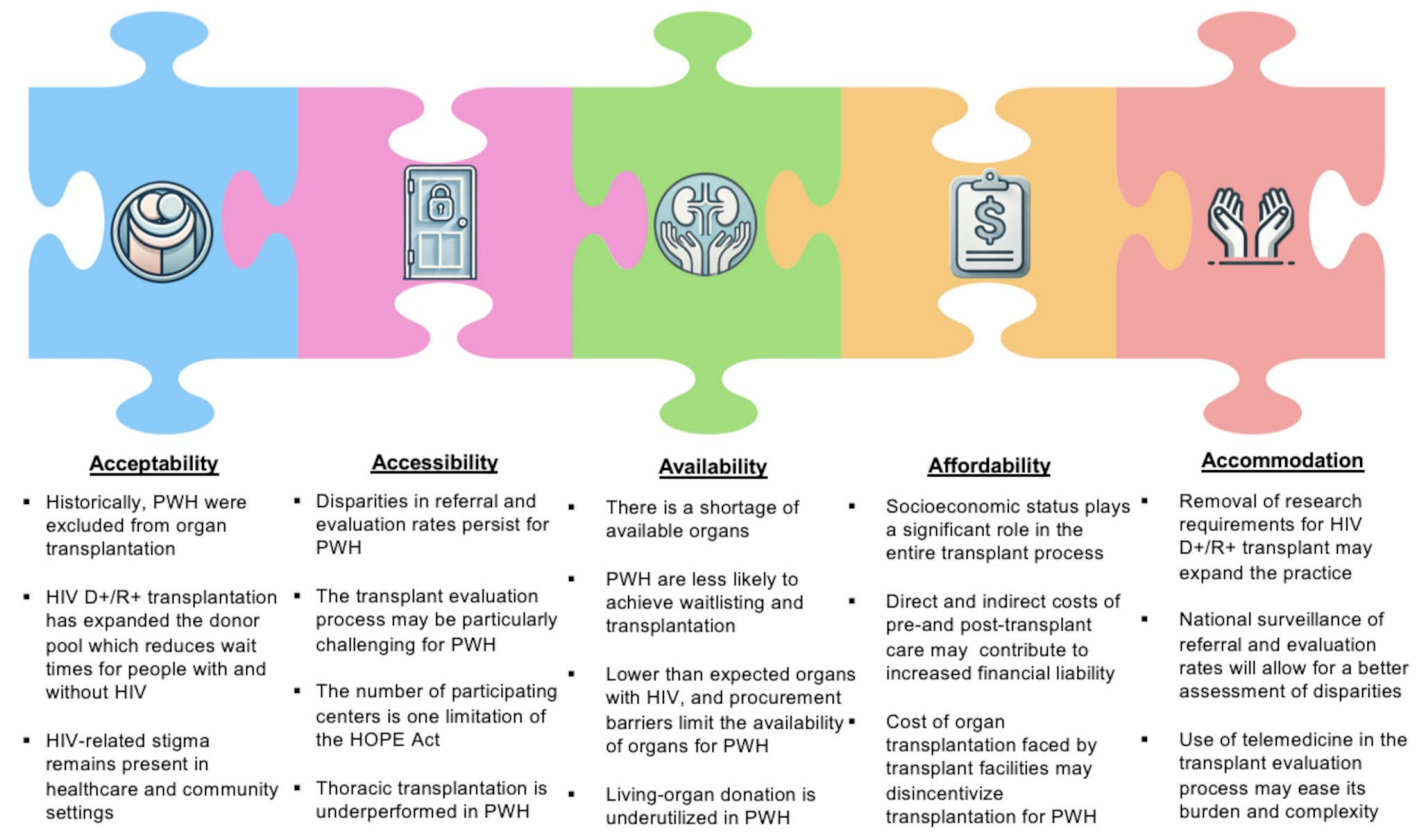
The Five A’s framework of assessing access to organ transplantation for persons with HIV (PWH)

Barriers to accessing organ transplantation for PWH are multifaceted. This review will examine the factors contributing to these disparities using a modified version of the Five A’s model: acceptability, availability, accessibility, affordability, and accommodation (Fig. 1) [9]. The original Five A’s framework was developed to describe healthcare providers’ and patients’ characteristics and expectations concerning healthcare access [9]. In this review, we will reinterpret these five dimensions in the context of access to organ transplantation, focusing on achieving equitable access for PWH.

## Acceptability

### Transplant Experiences among PWH

In the early 1990s, small “proof of concept” studies were performed to examine kidney transplantation in PWH. These studies suggested that PWH had worse outcomes, though these were before the advent of highly effective ART to treat HIV [10, 11]. These studies reinforced the notion that transplantation in PWH was not acceptable. In the late 1990s, a national survey revealed that 88% of the 148 responding transplant centers did not consider kidney transplantation appropriate for individuals with HIV [3]. These centers would not offer kidney transplantation to PWH and end-stage renal disease (ESRD), even if they were otherwise good candidates.

In 2003, Stock et al. published promising outcomes on PWH who underwent kidney and liver transplantation. The study included 14 PWH with CD4 counts > 200 for at least 6 months, no prior opportunistic infections, and undetectable HIV viral loads for at least 3 months for kidney transplant and predicted ability to achieve HIV suppression for liver transplant. At a mean follow-up of 480 days, 100% of PWH who received kidney transplants were alive with functioning grafts (*n* = 10), and 75% of liver transplant recipients were alive with functioning grafts (*n* = 4) [12], all without HIV disease progression.

The results of that pilot study led to a larger landmark study funded by the National Institutes of Health (NIH) that established kidney and liver transplantation as the standard of care for PWH [13]. The study included 150 kidney transplant recipients with HIV prospectively followed for a median of 1.7 years. The results demonstrated that patient and graft outcomes were better than those of older kidney transplant recipients (≥ 65 years) and equivalent to outcomes reported for the general population of kidney transplant recipients in the national registry [13]. Since then, the number of kidney transplants among PWH has significantly increased. In 2018, over 200 PWH received a kidney transplant [14]. There remains concern over increased rates of rejection among transplant recipients with HIV though, fortunately, this has not resulted in increased rates of graft loss or affected patient survival [13, 15, 16].

Similarly, the number of liver transplants performed in PWH has increased from 1 in 1996 to 63 in 2018, with non-viral liver disease now the leading cause of hepatic failure [17]. Survival of liver transplant recipients with HIV, especially those who were co-infected with hepatitis C virus (HCV), initially lagged behind populations without HIV [18–20]. However, more recent studies have demonstrated similar post-liver transplant outcomes among liver transplant recipients with and without HIV [16, 21]. These improved outcomes are likely driven by the advent of direct-acting antivirals (DAAs) for HCV treatment, improved HIV therapy, and more experience with performing transplants in this population. The success of organ transplantation in PWH has transitioned conversations from “whether” PWH should receive organ transplants to “which” PWH should receive organ transplants. It is now generally understood that PWH should not be excluded from being considered for organ transplantation simply because of their HIV status.

### HIV-to-HIV Transplantation

Another monumental shift in the history of solid organ transplantation for PWH occurred with the advent of transplantation from donors with HIV to recipients with HIV (HIV D+/R+). South Africa has one of the largest populations of PWH globally. Until 2009, the South African healthcare system provided dialysis solely as a temporary measure for patients awaiting kidney transplantation. However, PWH with ESRD were ineligible for kidney transplants, effectively excluding them from this life-saving intervention. As a result, PWH with ESRD faced exceptionally high mortality rates and nearly a third of available organs were discarded [22, 23]. In 2010, Muller et al. reported on early experiences with four HIV D+/R + kidney transplants. At 12 months post-transplant, all four patients exhibited good renal function, no significant graft rejection, and none required dialysis [24]. A subsequent study provided longer-term outcomes on 27 patients who had undergone HIV D+/R + kidney transplants. Patient survival rates at 1, 3, and 5 years were 84%, 84%, and 74%, respectively, while graft survival rates were 93%, 84%, and 84%, respectively [25].

These pioneering experiences paved the way in the United States for the HIV Organ Policy Equity (HOPE) Act in 2013, which permits HIV D+/R + organ transplantation under research protocols. This milestone not only expands the donor pool for PWH, but the overall expansion of the donor pool also benefits those without HIV on the organ transplant waitlist. Furthermore, it solidifies organ transplantation for PWH as morally and ethically acceptable [26].

### The Role of Stigma

Despite significant advances in the management of HIV and its related comorbidities, HIV remains a highly stigmatized condition, both within communities and healthcare systems. This stigma is a barrier to care for individuals seeking disease prevention services, treatment for acute or chronic conditions, or support in maintaining a healthy quality of life [27–29]. Over 25% of people with HIV in the U.S. report experiencing stigma within healthcare settings [30], and HIV-related stigma is particularly prevalent in the Deep South of the United States —Alabama, Georgia, Louisiana, Mississippi, South Carolina, and Tennessee [29]. Notably, these states account for more than 50% of new HIV diagnoses in the United States [31].

The field of organ transplantation is not without HIV-related stigma. A study assessing barriers to the implementation and expansion of the HOPE Act found that, while there was high awareness of the Act among organ procurement organizations (OPOs) and a consensus that HIV status should not influence donor selection, interview responses told a different story [32]. Some participants mentioned actively seeking out other exclusionary comorbidities to disqualify donors with HIV, and in some cases, there was notable reluctance among hospital staff to participate in the procurement process. Additionally, stigmatizing beliefs were observed among some healthcare workers, including assumptions that PWH are more likely to be homeless or that HIV remains a fatal disease [32]. Addressing and reducing HIV-related stigma will be essential in improving access to organ transplantation for PWH.

## Accessibility

### Barriers in the Transplant Process

PWH face several medical and logistical obstacles to be added to the waitlist for organ transplantation. In addition to meeting center-specific criteria, PWH are recommended to have a CD4 + count greater than 200 cells/µL and be virally suppressed for at least three months before transplantation [4]. For liver transplantation, the recommended CD4 + count threshold is > 100 cells/µL because splenomegaly can lower absolute CD4 T cell count and > 200 cells/µL for those with a history of an opportunistic infection [4]. PWH often experience delayed referrals and evaluations for organ transplantation. A study focusing on the steps to kidney transplantation for PWH in ESRD Network 6 revealed that PWH are less likely to be referred for kidney transplant, wait longer to begin the transplant evaluation process, and are less likely to be waitlisted [33]. In single-center studies, reasons that PWH were not referred to transplant services or were not considered an appropriate candidate included HIV antiretroviral resistance, active substance use, nonadherence (with ART, dialysis and/or the evaluation process), and HCV co-infection [34, 35].

Delays in referral to liver transplant can be detrimental as some studies have shown PWH with end-stage liver disease have an increased risk of pre-transplant death in HIV/HCV co-infected persons compared to persons without HIV despite having the same Model for End-Stage Liver Disease (MELD) score [36]. However, another study noted, after controlling for variables such as CD4 cell count and HIV viral load, MELD score was the only independent risk factor for pre-transplant mortality in liver transplant candidates with HIV [37]. As such, early and timely referral to liver transplant for PWH who are appropriate for transplant and have a MELD score ≥ 15 (predictive of a survival benefit from liver transplantation) should be placed on the waitlist and receive a liver transplant as soon as possible [21].

The most significant hurdles remain completing the transplant evaluation process and achieving waitlisting, as described in both national and single-center studies [7, 33, 34]. Racial disparities further compound these barriers. For example, a national study reported that Black individuals with HIV are 65% less likely to be added to the waitlist compared to White individuals without HIV [38]. Several factors likely contribute to the decreased rates of waitlisting among PWH. Prior studies have shown a high rate of failure to complete the necessary transplant evaluation workup, suggesting that the complexity of the process may be particularly burdensome for PWH [34, 35]. Social circumstances, such as economic and social conditions, likely play a significant role in a PWH’s ability to navigate complex healthcare systems [39–41] and often fragmented transplant process. When interviewed, PWH who were not engaged in HIV care cited issues like employment (e.g., having to take time off work without sick leave), lack of transportation, homelessness, and gaps in insurance coverage as reasons for gaps in care [42]. Unsurprisingly, these same challenges can hinder the ability of PWH to fully engage in the transplant evaluation process and navigate the numerous required appointments and procedures. Furthermore, suboptimal social support has been demonstrated to be associated with lower adherence to ART and poor engagement in HIV care [43]. This can be of concern because social support is a critical factor in determining transplant candidacy. In one study that interviewed transplant providers assessing waitlist suitability in the general population, social support was identified as the second most important factor in waitlisting decisions [44].

### Limited Access To HOPE

**Fig. 2:**
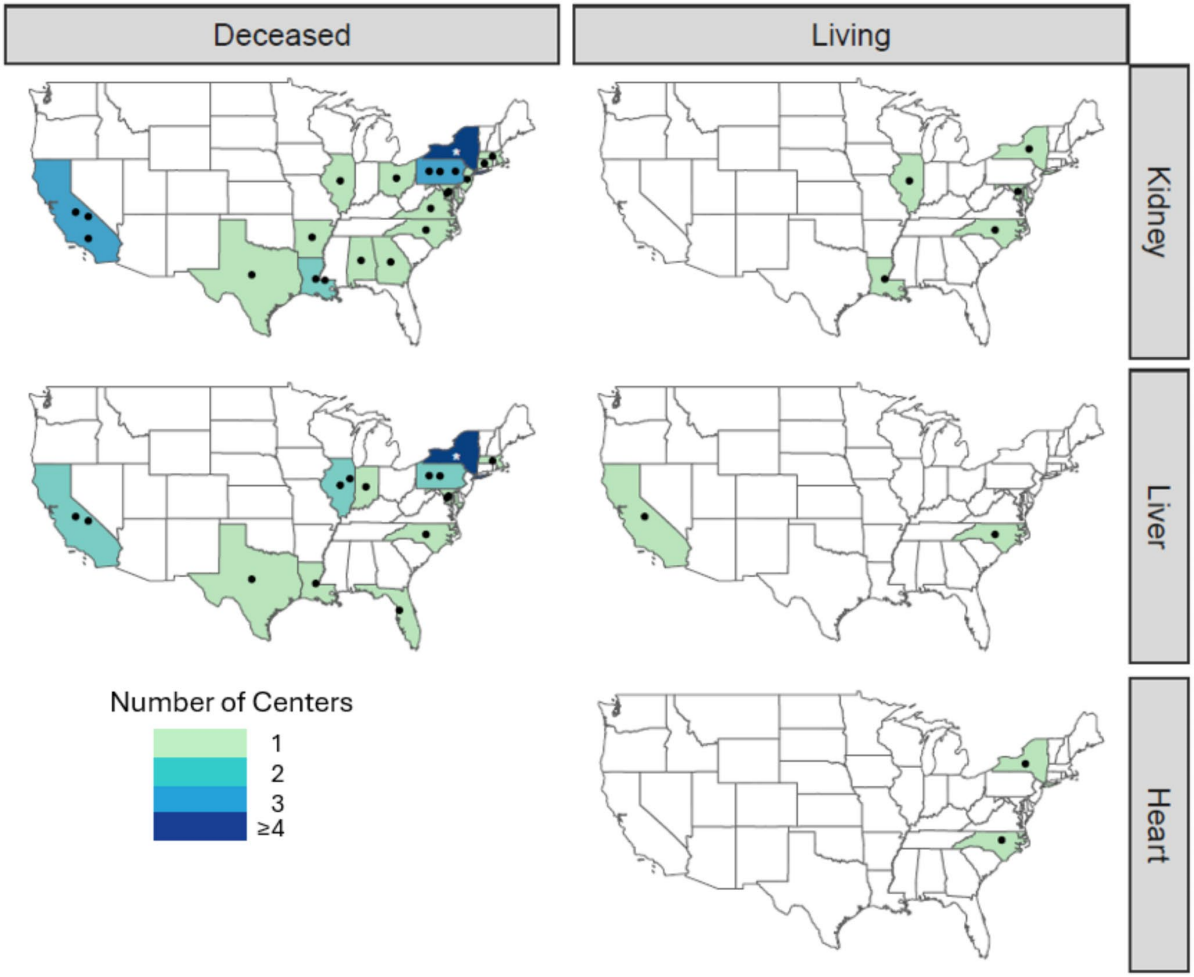
Transplant centers approved for HOPE Act. This figure depicts transplant centers approved to perform deceased kidney, liver and heart transplants and living kidney and liver transplants under the Organ Procurement and Transplantation Network HOPE Act Variance as of May 2024. The asterisk (*) in New York represents 5 centers in New York City

While the HOPE Act provides expanded access to transplantation for PWH, certain limitations must be acknowledged. Initial regulations for the HOPE Act mandate that HIV D+/R + transplants be conducted under protocols approved by an Institutional Review Board (IRB), with an Organ Procurement and Transplantation Network (OPTN) variance, and only if the transplant team meets specific experience criteria. Specifically, the combined experience of the transplant physicians and HIV physicians on the team must include at least five organ-specific cases over four years [45]. As of May 2024, 26 kidney transplant programs are approved to perform HOPE Act transplants, 19 programs are approved for liver transplants, and two programs are approved for heart transplants [45]. However, these institutions are not geographically evenly distributed. Figure 2 illustrates the distribution of transplant programs approved for HOPE transplants, with most centers clustered in the eastern United States. This concentration makes HOPE organs less accessible to PWH in the central and western regions of the country.

Despite the federal policy changes, state-level laws may still restrict some states from participating in HIV D+/R + transplantation. Three states currently prohibit organ donation from PWH under any circumstances, and other states may not address the issue at all [46]. However, it remains unclear how strictly these laws are enforced, as there have been no reported cases of PWH being prosecuted for donating organs. In states where explicit guidance on the use of organs from PWH is absent, the decision on how to interpret and proceed with HIV D+/R + transplantation is left to the discretion of Organ Procurement Organizations (OPOs), transplant centers, and the broader community. Nonetheless, 12 transplant programs within these states are approved to perform HIV D+/R + transplantation, suggesting that it is permissible in those locations [46]. Expanding the implementation of the HOPE Act will require continued advocacy and education of OPOs and transplant centers.

One unexpected advantage of HOPE Act is the discovery and use of organs from donors with false positive HIV screening tests. Previously these organs were discarded due to inadequate time for confirmatory testing and regulatory concerns. However, since HOPE, organs from these donors can be used easily for candidates with HIV within HOPE Act studies [47]. It is estimated that there will 50–100 HIV false-positive donors per year, allowing for even more organs to be accessible to PWH.

### Thoracic Transplantation

Accessing thoracic transplantation remains a significant challenge for PWH, and experience with heart and lung transplants has lagged behind that of abdominal organ transplants. One of the larger studies assessing outcomes of thoracic transplantation in PWH included 21 heart transplants, 7 lung transplants, and 1 heart-lung transplant. The one year survival rates were comparable to those reported in International Society for Heart and Lung Transplantation (ISHLT) registries for heart and lung transplants (90% for heart transplant recipients with HIV and 86% for lung transplant recipients with HIV) [48]. However, 5-year survival rates for heart transplant recipients with HIV were slightly lower than those for recipients without HIV. Another study on heart transplantation in PWH reported more promising outcomes, with patient survival rates of 100%, 88%, and 88% at 1, 3, and 5 years, respectively, and similar rates for graft survival [49]. These findings suggest that having HIV should not automatically exclude someone from being considered for heart transplantation. Despite these positive outcomes, many centers remain hesitant to perform these transplants in PWH [50].

Rouzaud et al. developed a questionnaire to assess the outcomes of lung transplantation in recipients with HIV across Europe. Data was collected from 25 transplant centers, resulting in one of the largest case series of lung transplants in PWH. Among the 22 patients included, post-transplant survival rates at 1, 3, and 5 years were 79%, 79%, and 79%, respectively. Acute cellular rejection occurred in 37% of cases, but at the last follow-up, 13 of the 22 patients remained fully active and independent [51]. These positive outcomes demonstrate that lung transplantation can be successfully performed in selected PWH with advanced lung disease. As experience with thoracic transplants in PWH continue to grow, it is hoped that more transplant centers will offer this option, thereby reducing the disparities in access to thoracic transplantation for PWH.

## Availability

### Burden of Organ Failure

Over 800,000 people in the US have ESRD, 69% of whom are on dialysis. Nearly 90,000 persons were on the kidney transplant waitlist in 2023. However, only 46,623 received a kidney transplant [52]. The etiology of ESRD in PWH includes both traditional risk factors such as hypertension, diabetes, and obesity, as well as HIV-related disorders, including HIV-associated nephropathy, renal tubular toxicity caused by antiretrovirals, immune complex kidney diseases, and co-infection with hepatitis B or C [53, 54]. PWH progress from chronic kidney disease to ESRD faster than the general population and are estimated to account for 1.5% of the dialysis population [55–57]. PWH who are of Black race experience the highest rates of ESRD [58].

Liver-related mortality is a leading cause of non-AIDS-related death among people with HIV (PWH), primarily due to decompensated cirrhosis and hepatocellular carcinoma (HCC) in those co-infected with hepatitis B virus (HBV) or HCV [59]. PWH are at a significantly higher risk of developing cirrhosis compared to individuals without HIV, and rates of liver decompensation are notably higher, especially among those triple-infected with HIV, HCV, and HBV [60]. One severe consequence of cirrhosis is the development of HCC, the incidence of which has been rising in PWH. A national study on trends in HCC incidence among PWH in North America found an increase from 0.28 to 0.75 cases per 1000 person-years between 1996 and 2015 [61].

The true demand for liver transplantation in the United States is difficult to quantify, as many people with liver failure are never added to the waitlist, and even fewer ultimately undergo transplantation. In 2022, over 50,000 people died from end-stage liver disease. In contrast, only 24,186 adult candidates were on the waiting list at any point during the year, and just 9,527 received liver transplants [62]. A recent study highlighted a 19.1% increase in liver-related mortality since 2018, with significant geographic variation. States with the highest liver-related mortality often had lower transplant rates, and 10 states had no liver transplant centers [63]. Among PWH, similar geographic disparities exist, with UNOS Region 3 (Alabama, Arkansas, Florida, Georgia, Louisiana, Mississippi, and Puerto Rico) performing the most liver transplants in PWH in 2011 while UNOS Region 6 (Alaska, Hawaii, Idaho, Montana, Oregon, and Washington) performed the fewest [64].

Despite the rising mortality from liver disease in the United States, as the indication for liver transplant shifts from viral hepatitis to metabolic-dysfunction-associated steatotic liver disease, the liver transplant waitlist is predicted to remain static over the next decade. This shift is expected to result in older candidates facing longer wait times, even with competitive MELD scores, and have a higher risk of waitlist dropout [65]. However, early waitlisting offers significant survival advantages for PWH. One study demonstrated that PWH on the liver transplant waitlist had a 68% survival benefit compared to those who did not undergo transplantation, underscoring the critical importance of timely access to liver transplant for this population [66].

### HIV D + Donor Pool

Undoubtedly, the demand for organ transplants far exceeds the supply of available organs. The availability of organs through the HOPE Act is constrained by the number of donors with HIV. Initial estimates suggested that the HOPE Act could provide 500–600 additional organs to PWH through the use of donors with HIV [67]. Furthermore, interviews with PWH revealed overwhelmingly positive views on HIV D+/R + transplantation, with many expressing a strong desire to become organ donors [68]. However, the actual donor pool has been significantly smaller than predicted. Contributing factors experienced by the patient may include historical restrictions on organ donation by PWH, lack of awareness that PWH can now donate organs, reluctance to discuss organ donation with family, ongoing stigma, and distrust of the healthcare system—particularly among Black individuals, who may face compounded barriers in transplantation [68, 69]. While organ procurement rates can, at times, be difficult to ascertain, a recent study prospectively characterized organ procurement for HIV D + referrals, procurement rates, and reasons for nonprocurement. They discovered that out of 710 HIV D + referrals, 24% had organs procured. Most commonly, medical reasons were listed as reasons for nonprocurement including organs from people with AIDS-defining infections (36%), and non-HIV related medical reasons consisted of organ failure (36%), high neurologic function (where the donor has high neurological function and is not expected to arrest in time) (31%), and cancer (14%) [70]. In 26% of cases, nonprocurement was due to nonmedical reasons such as no authorization (42%), no waitlist candidates (21%), or no transplant center interest (20%) [70]. These findings highlight the need for targeted strategies to address both medical and nonmedical barriers to organ procurement, ensuring that more potential HIV D + can contribute to the available organ pool.

### Living Organ Donation

Living kidney transplantation is the most common form of living organ donation. It offers several advantages, including a shorter waiting period, reduced risk of delayed graft function, a better potential match leading to a lower risk of rejection, and longer allograft survival [71]. These benefits extend to PWH as well. Stock et al. reported a significantly lower risk of graft loss in PWH who received a kidney from a living donor (HR 0.2; 95% CI 0.04 to 0.80) compared with those who received kidneys from deceased donors, alongside lower rates of rejection (15% vs. 46%) [13]. Despite these benefits, PWH are less likely to receive a living organ donation. Locke et al. found that the likelihood of living donor kidney transplantation among PWH was 47% lower (adjusted HR 0.53; 95% CI 0.44–0.64) compared with the non-HIV population [7].

The reasons for this disparity have not been thoroughly explored in the literature. One possible contributing factor is HIV-related stigma, which may limit individuals’ ability to maintain strong social relationships, ultimately shrinking the social networks of PWH [72]. Additionally, individuals within the social networks of PWH may face barriers to living organ donation, such as the potential for lost wages or even job loss during recovery, surgery-related expenses not covered by insurance, or the challenges of obtaining or affording insurance post-donation [73].

The option of HIV D+/R + transplantation could help increase living organ donation among PWH. In a survey, over 60% of respondents with HIV indicated a willingness to be living kidney donors [68, 74]. Their motivations included a desire to normalize living with HIV, reduce HIV-related stigma, and contribute to research [74]. Though limited, early experiences with HIV D+/R + living kidney transplantation have been promising. Durand et al. reported on the first three living kidney donors with HIV under the HOPE Act, with follow-up periods of two to four years. Donor outcomes were excellent with maintenance of HIV suppression, stable CD4 cell counts, and declines in estimated glomerular filtration rate (eGFR) as would be expected post-donation [75]. All three donors cited specific benefits and motivations for donating, such as autonomy and reducing HIV-associated stigma, and all three were authors of the study [69].

As with any living organ donor, living donors with HIV should be carefully selected. Studies have estimated that living kidney donors may have a slightly higher risk of developing ESRD post-donation [76] and may have comorbidities such as hypertension and diabetes, which can accelerate renal function decline after donation [75]. As experience with HIV D+/R + living organ donation grows, this approach could become a promising strategy to expand the pool of available organs for PWH.

Living donor liver transplant (LDLT) was started in the 1990s as a strategy to address the severe shortage of deceased donor organs and reduce waitlist mortality. However, utilization of this procedure in the United States remains low, given the potential for morbidity and possible mortality to the donor. As such, experiences with LDLT in PWH have been limited. In 2017, the first successful case of LDLT from a mother with HIV to her child who did not have HIV was reported in South Africa [77]. In 2023, a successful case of an HIV D+/R + LDLT from a spouse with HIV to a spouse with HIV/HCV coinfection was reported in Italy [78]. Expansion of LDLT in the United States among PWH will require several factors including transplant centers having the willingness to perform these transplants, increasing the living donor pool through educating the community and increasing awareness, and additional financial support for the donor [79].

## Affordability

### Cost of Transplantation

Despite the benefits of organ transplantation, the financial burden, particularly in the United States, where the healthcare system is dependent on insurance, cannot be ignored. A potential candidate’s socioeconomic status (SES) plays a role in every step of the process: the development of the underlying disease, the progression of the disease, the ability to complete the transplant evaluation process, and transplant outcomes [80–82]. It also influences the type of transplant achieved, with those of lower SES being less likely to receive a living organ donor [80].

Organ transplantation is often cost-effective compared to the management of end-stage organ diseases [83–86]. Kidney transplantation, in particular, is cost saving compared to dialysis [87]. Candidates for HIV D+/R + transplantation benefit from shorter kidney transplant wait times at a median of 10.3 months compared to 60.8 months for HIV D- candidates [88], further adding to the cost savings of HIV D+/R + transplantation. However, the indirect costs that accrue throughout the transplant process can be challenging to quantify. Contributing factors to financial hardships for candidates and caregivers include pre-transplant hospitalizations, needing to take time off work to attend numerous evaluation appointments resulting in lost wages, transportation, lodging, parking fees, and childcare expenses [89, 90].

For those who successfully undergo transplantation, the costs of post-transplant care can be substantial. In one study that evaluated post-liver transplant liability (what a patient owed), financial liability ranged from 3% of individuals having no liability to 21% having extreme liability defined as owing >$10,000 for 1-year post-liver transplant care [91]. The cost of immunosuppressant medications can add to the financial instability of transplant recipients. Medicare covers 80% of immunosuppressant medication costs for the first three years post-transplant and private insurance typically covers the remainder. Despite this, many transplant recipients may still struggle to afford post-transplant medications. In a study by Gordon et al., 29% of kidney transplant recipients reported difficulties in affording transplant-related medications and leisure activities, housing, vehicles, and other essentials of daily living [92]. While the financial burden of organ transplantation is not unique to PWH, this population may be particularly vulnerable to the economic hardships of organ transplantation. Addressing these financial burdens is crucial to improving long-term outcomes and quality of life for all organ transplant recipients, particularly for those who are at an economic disadvantage.

Another consideration is the financial burden placed on transplant facilities to support a growing transplant program. While organ transplantation is cost-effective over time, the initial expenses associated with transplantation are substantial and absorbed by the transplant facilities. Cheng et al. reported that the annual Organ Acquisition Cost Center (OACC) cost per kidney transplant increased from $81,000 in 2012 to $100,000 in 2017 [93]. Factors such as larger transplant waitlists and the higher burden of patient comorbidities were significantly associated with increased OACC costs per transplant. These rising costs may discourage transplant centers from aggressively pursuing transplants for populations that may be at higher risk for post-operative complications, including PWH, thereby exacerbating existing disparities in access to transplantation.

## Accommodation

### Expanding HOPE

Efforts to better accommodate PWH within the organ transplant system should be carefully evaluated. A key approach is transitioning HIV D+/R + transplantation from research protocols to a standard practice of care. Under the original HOPE Act Safeguards and Research Criteria, transplant centers interested in performing HIV D+/R + organ transplants were required to perform these transplants only with approved Institutional Review Board (IRB) research protocols and to have performed at least five transplants of the same organ type in PWH within the preceding four years [45]. The latter requirement is particularly restrictive for pancreas and thoracic transplants, given the lower volume of these procedures, and has hindered the expansion of HOPE [94]. A recent ruling from the Department of Health and Human Services (HHS) has lifted the clinical research requirements for transplanting HIV D + kidneys and livers [95]. Lack of safety data for HIV D+/R + lung, pancreas, islet and intestine limited the ruling only to kidney and liver HOPE Act transplants. This revolutionary advancement in HIV D+/R + kidney and liver transplants will expand the availability of and access to the HIV D + organ pool, help reduce stigma, and thereby lessen health disparities in abdominal organ transplantation.

### National Surveillance Systems for Referral and Evaluation

National surveillance data currently focus solely on tracking metrics from the waitlisting stage onward. While policies such as restructuring payment models for dialysis facilities and nephrologists to incentivize waitlisting and transplantation aim to mitigate inequities [96], disparities often emerge much earlier in the process—beginning with referral [33]. This underscores the critical need for national surveillance systems to monitor not just waitlisting and transplant rates but also referral and evaluation stages. A lack of comprehensive or transparent data allows these disparities to persist unaddressed. Gaining a deeper understanding of how PWH navigate the entire transplant process is essential for identifying and addressing barriers to transplantation, ultimately improving equity in access to life-saving procedures.

### Utilization of Telemedicine

The COVID-19 pandemic highlighted the necessity for more adaptable approaches to the transplant evaluation process. Integrating telehealth into transplantation workflows offers a promising solution to alleviate the geographic, financial, and logistical barriers faced by patients, barriers that are particularly pronounced for PWH. At a U.S. Veterans Affairs transplant center, telemedicine use during pre-transplant evaluations significantly reduced costs and improved timeliness, enhancing access to transplantation [97]. Similarly, another study demonstrated that leveraging telemedicine to initiate kidney transplant evaluations improved communication with community providers and nephrologists, markedly increased evaluation completion rates for African American kidney transplant candidates, and increased the number of kidney transplants performed in African American candidates by 15% [98]. These findings underscore the potential of telemedicine to streamline the transplant evaluation process, reduce inequities, and improve access to transplantation. For PWH, who already face unique challenges in accessing transplantation, expanded telehealth adoption could be transformative in addressing systemic disparities and enhancing equitable care delivery.

## Conclusion

As the prevalence of comorbidities among PWH increases, the rates of organ failure are expected to rise as well. Although PWH have comparable transplant outcomes to the general population, they continue to face significant barriers to accessing organ transplantation. By applying the Five A’s framework, we have identified the complex, multi-level obstacles to organ transplant faced by PWH. Factors related to patients, transplant systems, and policies all contribute to the challenges encountered throughout the transplant process. Improving tracking of each step in the transplant journey, reducing regulatory barriers for the expansion of HIV D+/R + transplantation, and increasing the use of telemedicine are crucial steps toward addressing the inequities that PWH face in accessing organ transplantation.

## Key References

- Stock PG, Barin B, Murphy B, Hanto D, Diego JM, Light J, et al. Outcomes of kidney transplantation in HIV-infected recipients. N Engl J Med 2010;363:2004–14. 10.1056/NEJMoa1001197.
-
  - This landmark paper established kidney and liver transplantation in PWH as safe and feasible.
- Muller E, Kahn D, Mendelson M. Renal Transplantation between HIV-Positive Donors and Recipients. N Engl J Med 2010;362:2336–7. 10.1056/NEJMc0900837.
-
  - This manuscript describes the first HIV D+/R + kidney transplants which were performed in South Africa.
- Locke JE, Mehta S, Sawinski D, Gustafson S, Shelton BA, Reed RD, et al. Access to Kidney Transplantation among HIV-Infected Waitlist Candidates. Clin J Am Soc Nephrol 2017;12:467–75. 10.2215/CJN.07460716.
-
  - This paper highlighted that nationally, PWH experience barriers to kidney transplantation.
- Blumberg EA, Rogers CC. Solid organ transplantation in the HIV-infected patient: Guidelines from the American Society of Transplantation Infectious Diseases Community of Practice. Clinical Transplantation 2019;33:e13499. 10.1111/ctr.13499.
-
  - Guidelines that inform practices surrounding solid organ transplantation in PWH.
- Durand CM, Zhang W, Brown DM, Yu S, Desai N, Redd AD, et al. A prospective multicenter pilot study of HIV-positive deceased donor to HIV-positive recipient kidney transplantation: HOPE in action. Am J Transplant 2021;21:1754–64. 10.1111/ajt.16205.
-
  - This multisite study was the first to report on favorable outcomes of HOPE HIV D+/R + transplantation.
- Durand CM, Massie A, Florman S, Liang T, Rana MM, Friedman-Moraco R, et al. Safety of Kidney Transplantation from Donors with HIV. New England Journal of Medicine 2024;391:1390–401. 10.1056/NEJMoa2403733.
-
  - This study solidifies that HIV D + transplantation is noninferior to HIV D- transplantation.

## Data Availability

No datasets were generated or analysed during the current study.
